# Author Correction: Accurate medium-range global weather forecasting with 3D neural networks

**DOI:** 10.1038/s41586-023-06545-z

**Published:** 2023-09-14

**Authors:** Kaifeng Bi, Lingxi Xie, Hengheng Zhang, Xin Chen, Xiaotao Gu, Qi Tian

**Affiliations:** Huawei Cloud, Shenzhen, China

**Keywords:** Computer science, Atmospheric dynamics

Correction to: *Nature* 10.1038/s41586-023-06185-3 Published 5 July 2023

In the version of the article originally published, there was an error in the Methods section “Mathematical settings”, where “*N*_lon_” and “*N*_lat_” were erroneously swapped in the text now reading “This is a 3D matrix of size *N*_lon_ × *N*_lat_ × 69, where *N*_lon_ = 1,440 and *N*_lat_ = 721 are the spatial resolution along the longitude and latitude axes”. This has now been corrected in the HTML and PDF versions of the article.

